# Spectral level repulsion and Lifshitz-like states in hyperuniform disordered photonic networks

**DOI:** 10.1038/s41377-026-02335-0

**Published:** 2026-05-20

**Authors:** Nicoletta Granchi, Gabriele Calusi, Kris Stokkereit, Matteo Lodde, Camilla Gonzini, René P. J. Van Veldhoven, Andrea Fiore, Marian Florescu, Francesca Intonti

**Affiliations:** 1 Department of Physics and Astronomy and LENS, University of Florence, Firenze, Italy; 2 Advanced Technology Institute and Department of Physics, University of Surrey, Guildford, UK; 3 Department of Applied Physics and Science Education, Eindhoven University of Technology, Eindhoven, The Netherlands; 4 Optoelectronics Research Centre, University of Southampton, Southampton, UK

**Keywords:** Photonic crystals, Nanocavities, Nanophotonics and plasmonics, Sub-wavelength optics

## Abstract

Disorder is emerging as a design principle in nanophotonics, offering new ways to control light beyond the limits of periodic architectures. Hyperuniform disordered networks, positioned between order and randomness, offer a unique platform for this exploration. Using large-scale numerical simulations and hyperspectral near-field imaging, we uncover spectral level repulsion among delocalized modes – a universal signature of interacting states in complex systems, from nuclear physics to quantum chaos, experimentally observed here for the first time in correlated disordered photonics. Within the same platform, we provide a phenomenological identification of classes of localized modes in hyperuniform disordered networks: genuine Anderson states and defect-induced resonances associated with Lifshitz-like photonic states. Unlike their electronic counterparts, these Lifshitz-like states arise from architecture-encoded topological defects and can hybridize into coupled “photonic molecules”. Our results reveal a potentially reconfigurable regime of disorder where localization, correlations, and coupling can be engineered, opening new opportunities for random lasing, optical filtering, and quantum photonics.

## Abstract (web summary)

Hyperuniform disordered photonic networks enable controlled light localization and spectral correlations, revealing level repulsion and Lifshitz-like states with tunable coupling, opening new possibilities for photonic devices and quantum technologies.

## Graphical abstract

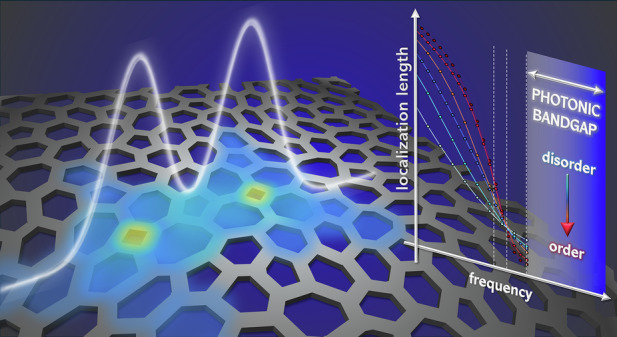

## Introduction

Localization is a fundamental phenomenon and a longstanding subject of research across many areas of physics, including electronics, photonics, and quantum optics^1–6^. In photonic systems, disorder is no longer regarded merely as a limiting factor, but increasingly as a resource for engineering novel light–matter interactions^7^. Anderson localization was first introduced in electronic systems to understand the suppression of electron diffusion in disordered solids due to interference from multiple scattering events^1^. This phenomenon, central to understanding metal-insulator transitions, has been extended to photonic systems: indeed, light can similarly undergo localization in disordered optical media, with interference effects preventing its propagation through the medium^2^. Given the growing reliance on photons as information carriers in advanced communication systems, these novel control mechanisms are of increasing scientific and technological importance. The photonic analog of Anderson localization enables novel ways to control light transport, but it also remains a central challenge in disordered photonics, with implications ranging from on-chip light management to quantum information processing^2–6^. In the last decade, hyperuniform disordered (HuD) photonic systems^8^ have emerged as a powerful framework in this scenario, with immense potential for both fundamental physics and applications. HuD geometries, laying at the interface between order and disorder, are notable for their suppressed long-range density fluctuations, and have showcased how carefully engineered disorder can manifest in exotic optical phenomena, from optical transparency^9^ and large photonic band gaps^10,11^ in the absence of periodic order, to robust light confinement^12^ and freeform waveguiding^13^ with rich modal statistics^14–17^. These unique structures exemplify how disorder can be engineered to control both spectral and momentum characteristics of light, and they have emerged as a promising nanophotonics platform with strategic advantages for applications such as quantum cascade lasing^18^ and enhanced light harvesting^19^.

Previous experimental studies have revealed the transition from light localization to diffusive transport in optically active HuD platforms^12,16,17^, showing that these correlated disordered systems host Anderson-like modes that are highly promising not only for fundamental studies but also for technological applications in light–matter interaction. The coexistence of deterministically tailored localized and diffusive transport within the same photonic material^10,12,16,17^ has therefore raised novel and important issues. In this work, we address three of these major issues.

We first examine the delocalized modes of the hyperuniform disordered network structures to determine whether their spectral statistics are uncorrelated or instead exhibit correlations characteristic of extended states. Level repulsion^20–25^ provides a universal diagnostic in this context: when modes overlap spatially, their eigenfrequencies cannot cross, and they exhibit spectral level repulsion, while localized modes remain uncorrelated. By exploiting the subwavelength spatial resolution of Scanning Near-field Optical Microscopy (SNOM)^12,16^ and analyzing the data via an autocorrelation approach^22,26^, we provide direct experimental evidence of level repulsion among delocalized modes of HuD systems, a universal signature of spectral correlations typically associated with chaotic or complex systems^27,28^ and now finally unveiled in a correlated-disorder photonic platform.

We next turn to the physical origin of localized modes, highlighting a fundamental distinction in electronic systems yet often neglected in photonics: localized states need not always be of the Anderson type. Electronic systems also host Lifshitz states^29–32^: rare, tightly confined resonances that emerge near the band-edge due to disorder. Distinguishing interference-driven Anderson states from disorder-induced Lifshitz states is fundamental in condensed matter physics as much as it is for photonics. Yet in the latter, this separation has often been overlooked, and direct experimental evidence of photonic Lifshitz states has so far been missing. Previous theoretical and numerical studies have already proposed their role in photonic systems^30–33^, also dealing with correlated disorder, to showcase theoretically how hyperuniformity can give rise to more than one class of localized photonic states. Ref. ^11^ distinguished two localization regimes in structurally correlated disordered photonic systems using transport simulations, identifying Anderson localization and a band-edge “pseudo-tunneling” regime.

On the experimental side, García et al.^34^ combined numerical simulations and photoluminescence experiments in one-dimensional photonic-crystal waveguides subject to weak fabrication-induced disorder to identify two distinct disorder-induced localization regimes. Still, unambiguous experimental identification of Lifshitz-like states has remained elusive, leaving a gap in the correct classification of localization phenomena. Here, we adopt an eigenmode-based approach and refer to the corresponding defect-associated band-edge states as Lifshitz-like, highlighting their spectral origin rather than their transport properties^11^. By introducing a rigorous statistical and experimental framework that overcomes the limitations of traditional level-spacing approaches^24^, we show that two-dimensional hyperuniform disordered slab networks host not only genuine Anderson-localized modes but also defect-induced resonances associated with specific topological features of the underlying hyperuniform architecture, such as four-sided cells^10,12,16^. We further show that these resonances are consistent with photonic Lifshitz-like states. Unlike their electronic counterparts, which are associated with statistical anomalies of exponentially small weight, photonic Lifshitz-like states in HuD are rooted in predictable topological defects, making their spatial and spectral location reproducible once the structure is defined.

This result directly informs the third aspect examined in this study. We show that, within an intermediate range of disorder, Lifshitz-like states are not isolated but can hybridize^3,4,35^ when sufficiently close in both space and frequency. Their coupling gives rise to “photonic molecules” with bonding and antibonding modes^35^, representing a minimal hopping scenario that bridges individual localization and collective interaction. Here, we report a direct demonstration of photonic Lifshitz-like states hybridizing into photonic molecules, establishing a correlated-disorder platform for mode coupling based on architecture-encoded defect physics. Importantly, we demonstrate this effect in a 2D photonic slab operating in TE polarization, which is the configuration most compatible with semiconductor technology. These theoretical and experimental findings pave the way for programmable cooperative pathways for light propagation through the disordered structure, like necklace states^3,36^.

## Results

**Fig. 1:**
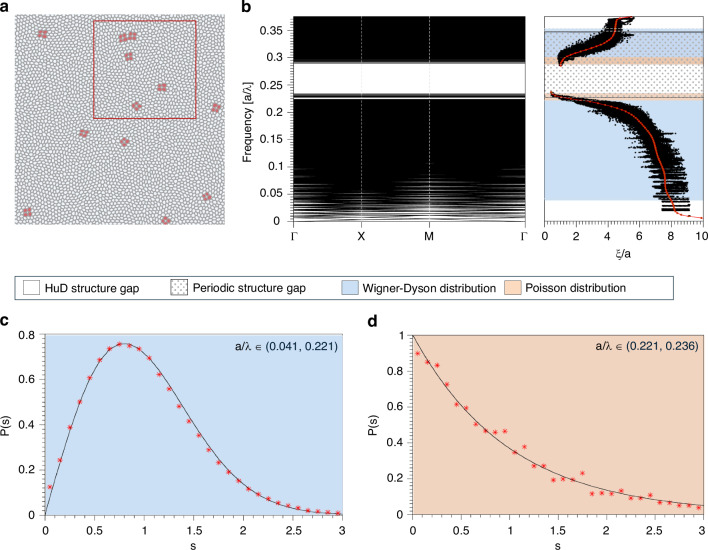
HuD network design, photonic band structure and energy-level statistics. **a** Sketch of the 24 × 24 μm^2^ 2D theoretical design of the HuD network with *N* = 4000, length scale *a* = 380 nm, wall thickness *u* = 0.34*a* and stealthiness *χ* = 0.5. **b** Left panel: 2D photonic band structure, with *a*/*λ* is the dimensionless frequency, and the high-symmetry directions in the reciprocal space defined by the irreducible Brillouin zone of the hyperuniform disordered sample. Right panel: localization length *ξ* of the HuD structure as a function of frequency, with the photonic band gap region of the corresponding honeycomb periodic structure (at the same filling fraction) highlighted as a dotted area. The spectral regions for which the level statistics are best fit by the Poisson distribution are shaded in light orange, while the ones for which the level statistics are best fit by the Wigner–Dyson distribution are shaded in light blue. The plot displays the calculated localization length for 50 samples and 17 k-points per sample (in black). The red curve represents a spline fit of the average value $\langle \xi /a\rangle$ in a narrow frequency range *Δ*[*a*/*λ*] = 5 × 10^−3^ around the frequency *a*/*λ* considered. The white region in the middle refers, accordingly with the left panel of (**b**), to the photonic band gap of the HuD pattern for *χ* = 0.5. **c** Level-spacing distribution for modes with frequencies in the lower blue region of the right panel of (**b**) and corresponding Wigner–Dyson distribution. **d** Level-spacing distribution for modes with frequencies in the lower orange region of the right (**b**) and corresponding Poisson distribution

The samples investigated in this work are based on the design of a purely two-dimensional stealthy hyperuniform disordered dielectric network, shown in Fig. 1a, generated using the tessellation protocol described in ref. ^10^ (see “Materials and methods” section for details). Stealthy hyperuniform structures^37,38^ are disordered point patterns whose structure factor vanishes exactly within a finite region of reciprocal space, i.e., $S\left({\bf{k}}\right)=0$ for ${\rm{| }}{\bf{k}}{\rm{| }} < {k}_{{\rm{C}}}$, where *k*_*C*_ is a cutoff wavenumber. The stealthiness parameter *χ* quantifies the fraction of constrained wavevectors and controls the strength of long-range correlations. In the large-*χ* limit, stealthy hyperuniform patterns approach crystalline order, whereas small *χ* corresponds to reduced structural constraints, and hence more disordered, hyperuniform structures^8,10^. Analogously to the lattice constant in photonic crystals, a length scale $a=L/\sqrt{N}$ is defined, such that an *N*-point hyperuniform pattern in a square box of side length *L* has a scatterer density of $1/{a}^{2}$ (here we use the stealthiness parameter χ = 0.5^10^, *N* = 4000, and *a* = 380 nm). The photonic band structure of the sample for transverse-electric (TE) polarization is calculated using a supercell approach, exploiting the fact that the HuD pattern is generated under periodic boundary conditions, and is shown in the left panel of Fig. 1b. The resulting band structure exhibits a photonic band gap of approximately 23% of its central frequency.

It has been previously demonstrated that the interplay of hyperuniformity, short-range geometric order, and uniform local topology results in the formation of a complete PBG^10^; its formation is directly linked to the Mie-like resonances^10,39–41^ of the individual scattering centers (for transverse magnetic, TM, polarization) or scattering cells (TE polarization) within the structure, when the scattered field is out of phase with the incident field, resulting in the absence of propagation channels for light^42^. Firstly, we numerically characterize the localization properties of the modes through the average localization length *ξ* (defined as in ref. ^24^ by *ξ* = (1/2)$\sqrt{1/{IPR}}$ with ${IPR}$ the Inverse Participation Ratio) at different frequencies, obtained by evaluating the average intensity distributions of 50 different realizations. Due to the size of the computational problem, we limit the localization length analysis to smaller samples consisting of 500 points each. The right panel of Fig. 1b reports the localization length *ξ* of the HuD structure as a function of frequency, with the photonic band gap region of the corresponding honeycomb periodic structure (at the same filling fraction) highlighted by the dotted area. This plot confirms that in HuD structures, low-frequency modes below the band gap have extended spatial profiles (larger *ξ*) that indicate a diffusive regime, while at higher frequencies closer to the photonic band edges, the modes become much more localized (smaller *ξ*)^16^.

A central challenge in disordered photonics is distinguishing between localized and delocalized states, i.e., establishing a quantitative measure to reliably classify optical modes according to their spatial extent. Spectral level repulsion is a universal signature of modal overlap^20–23,27^: the link between modes spatial localization and their eigenfrequency is at the basis of the level repulsion phenomenon, as it is directly related to the spatial extension of the photonic modes present in the structure; for modes whose spatial extent covers a large fraction of the sample, overlap is unavoidable and in order to maintain orthogonality the modes “repel” each other in the frequency domain and hence need to have different frequencies^24^. We therefore approach the distinction between localized and delocalized modes with numerical simulations in 2D through the level-spacing statistics method^24,25,27^. Defining ${s}_{n,k}=\frac{\Delta {\omega }_{n}(k)}{\left\langle \Delta {\omega }_{n}(k)\right\rangle }$ as the dimensionless spacing between adjacent bands *n* and *n* + 1, with ${\mathrm{\varDelta \omega }}_{n}\left(k\right)={\omega }_{n+1}\left(k\right)-{{\rm{\omega }}}_{n}(k)$, the delocalized mode spacings are characterized by the Wigner–Dyson distribution^24,28^

1 $$P\left(s\right)=\frac{{\rm{\pi }}s}{2}\,exp\left(-\frac{{\rm{\pi }}{s}^{2}}{4}\right)$$

for which $\mathop{lim}\limits_{s\to 0}P\left(s\right)\to 0$. In contrast, localized modes with field distribution confined to disjoint regions of the sample are spectrally uncorrelated. The mode's level statistics are therefore characterized by a Poisson distribution,

2 $$P\left(s\right)=exp\left(-s\right)$$

and the modes can have arbitrarily close frequencies since the level repulsion is absent: $\mathop{lim}\limits_{s\to 0}P\left(s\right) > 0$. To identify the spectral ranges over which the level-spacing statistics are best described by either a Poisson distribution or a Wigner–Dyson distribution, we perform a global search for chi-square minimization of the respective distributions. We find that regions located at the band-edge for $a/{\lambda }\in \left(0.221,\,0.236\right)\cup \left(0.284,\,0.304\right)$ (highlighted in light orange in the right panel of Fig. 1b), the level spacing is best described by a Poisson distribution (see Fig. 1d), whereas for $a/{\lambda }\in \left(0.041,\,0.221\right)\cup \left(0.304,\,0.353\right)$ (highlighted in light blue in the right panel of Fig. 1b), the level statistics is best described by the Wigner–Dyson distribution (see Fig. 1c). We note that the level-spacing analysis is performed over finite frequency intervals, whose boundaries are systematically varied to identify the ranges best described by Wigner–Dyson or Poisson statistics. As a result, the assigned statistics represent dominant spectral regimes rather than individual modes, and Wigner–Dyson behavior may persist even when the average localization length within an interval remains only a few times the characteristic length scale *a*.

Interestingly, the Wigner–Dyson level-spacing statistics observed for delocalized modes correspond to the Gaussian Orthogonal Ensemble (GOE) of random matrix theory, the appropriate universality class for time-reversal-symmetric wave systems (see, e.g., ref. ^28^). Beyond the familiar level repulsion behavior at small spacings, GOE spectra exhibit pronounced spectral rigidity, whereby fluctuations in the number of levels within large frequency intervals are strongly suppressed relative to uncorrelated (Poisson) sequences. In the language of point processes, this suppression of long-wavelength spectral fluctuations is the spectral analog of hyperuniformity: GOE eigenvalue sequences are themselves hyperuniform in one dimension^8^. This correspondence points to a close connection between the structural correlations of stealthy hyperuniform media and the long-range correlations emerging in their mode spectra. At the same time, real-space hyperuniformity alone does not imply GOE statistics; rather, GOE-like behavior arises from significant modal overlap and interaction, consistent with the delocalized transport regime identified here.

**Fig. 2:**
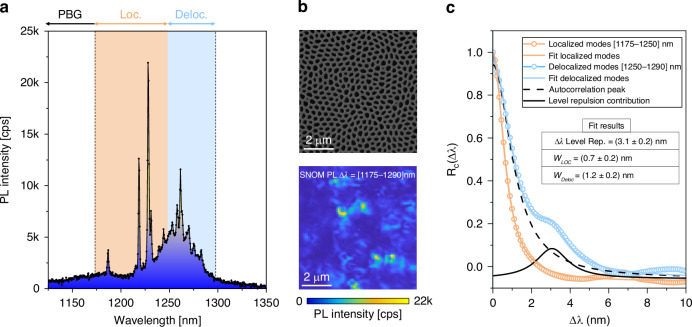
Experimental observation of spectral level repulsion through SNOM hyperspectral imaging. **a** Typical SNOM PL Intensity spectrum acquired in a single position of the tip on the HuD sample; the spectrum is divided into three regions, highlighting the PBG, the localized modes, and the delocalized modes. **b** Upper panel: SEM image of a detail of the patterned membrane. Bottom panel: SNOM PL collective map filtered around the spectral window (1175–1290 nm) displaying the spatial distribution of Anderson resonances on the sample. **c** Autocorrelation function *R*_*c*_ (∆*λ*) evaluated in the spectral windows corresponding to the localization (light orange curve, (1175–1250 nm)) and delocalization regimes (light blue curve, (1250–1290 nm)). The sampling over ∆*λ* is 0.11 nm, corresponding to the spectral resolution of the setup. The two curves are fitted respectively with a Lorentzian (orange) and a sum of two Lorentzian peaks (blue)

We experimentally investigate these two regimes in HuD structures by exploiting the subwavelength resolution of SNOM to resolve the spectral and spatial structure of delocalized modes of samples realized with the optically active slab technology^12,16,17^: a 8 × 8 μm^2^ crop of the HuD pattern (highlighted by the red rectangle in Fig. 1a) was employed to pattern a GaAs 220-nm-thick membrane with InAs quantum dots embedded in the middle. More details on the sample fabrication and SNOM technique can be found in the “Materials and methods” section. In the following, we focus on dielectric modes below the PBG and across the lower band-edge. In Fig. 2a it is reported a typical SNOM PL spectrum acquired at a fixed position on the structure, whose details can be observed in the Scanning Electron Microscopy (SEM) image (top panel of Fig. 2b). The spectrum exhibits a high density of peaks of different intensity and spectral width, corresponding to different light transport regimes^16^, coherently with the theoretical results shown in Fig. 1: after the PBG region, dielectric localized modes with sharp peaks and high-quality factors arise (light orange area). Farther from the PBG edges, broader resonances with delocalized spatial distributions appear, indicating a diffusive regime (light blue area). The hyperspectral SNOM Photoluminescence (PL) map filtered around the entire spectral window ∆*λ* = [1175–1290] nm allows to clearly visualize the collective spatial distributions of the dielectric modes at the PBG lower edge (Fig. 2b lower panel). Since the contribution of level repulsion to macroscopic spectra is very weak, if not absent, we leverage the spatially resolved SNOM technique, which selectively excites only modes close to each other in real space, enabling a direct insight into this universal signature of mode spatial overlap. Moreover, given the finite dimension of the sample and therefore the limited number of modes supported by the physical structure, to experimentally detect the level repulsion phenomenon, we do not rely on the level-spacing statistics approach^24^ that requires a large number of modes to guarantee reliable results. Instead, we evaluate the autocorrelation function of the experimental near-field spectra, which is expected to be strongly influenced by level repulsion features for small spectral separations (∆*λ*)^22,26^. Indeed, the autocorrelation function *R*_*c*_ (∆*λ*) represents a microscopic statistical property of the system and can be evaluated by averaging the autocorrelation of the single spectra collected in every pixel of the SNOM hyperspectral map (Fig. 2b bottom panel) and subtracting the uncorrelated contribution given by the autocorrelation of the average of all the spectra in the map. The full procedure is described in refs. ^22,26^ and summarized in Section S1 of the Supplementary Information. The autocorrelation function constitutes a different observable from the nearest-neighbor spacing distribution: for Wigner–Dyson statistics, level repulsion implies that the two-level correlation function vanishes at zero separation. In an idealized system of infinitely sharp spectral lines, this would manifest as a suppression of correlations, ${R}_{c}\to 0$, as $\Delta \lambda \to 0$, corresponding to a dip in the autocorrelation function at small spectral separations. In practice, each discrete energy level has a finite Lorentzian linewidth, and the self-correlation of these Lorentzians generates a pronounced peak in $R(\Delta \lambda )$ at $\Delta \lambda =0$, as shown in previous studies^22,26^. As a result, when reconstructing ${R}_{c}(\Delta \lambda )$ experimentally, the ideal dip associated with level repulsion does not appear as a zero at $\Delta \lambda =0$, but instead emerges as a characteristic shoulder or minimum at a finite Δ*λ*. The position of this feature is determined by the interplay between the mean level spacing and the Lorentzian linewidth. By contrast, for spectra obeying Poisson statistics, where level repulsion is absent, ${R}_{c}(\Delta \lambda )$ does not exhibit such short-range suppression and is dominated solely by the central peak.

In our sample, ${R}_{c}(\Delta \lambda )$ must be evaluated in two different spectral windows, given the different localization length of the modes sustained by the sample. In Fig. 2c we report the two curves of ${R}_{c}(\Delta \lambda )$ calculated within the localized modes spectral window (1175–1250 nm) (light orange curve) and delocalized modes (1250–1290 nm) (light blue curve). For more details on the data processing methods and criteria of choice of spectral intervals, see Section S1 of Supplementary Information. For both cases, we observe the autocorrelation peak that occurs for the self-correlation of each spectrum (∆*λ* = 0 nm). This broadened peak weakens the level repulsion dip into a shoulder, clear in the light blue curve and centered at ∆*λ* = (3.1 ± 0.2) nm as extracted by the fit. In contrast, the autocorrelation of localized modes does not exhibit any trace of level repulsion, proving that they are uncorrelated. Interestingly, our technique provides an additional physical insight into the photonic features of modes. The autocorrelation peak is narrower for localized modes characterized by higher quality factors, *Q*, than the delocalized ones (*Q* is defined as the ratio between the resonance wavelength and its linewidth, $Q={\lambda }_{0}/w$, and hence quantifying the lifetime of an optical mode). It can indeed be used to indirectly measure the typical spectral width of the modes in the two regimes; the values extracted from the fits result in *w*_*Loc*_ = (0.7 ± 0.2) nm and *w*_*Deloc*_ = (1.2 ± 0.2) nm, in excellent agreement with the previously reported Q-factor theoretical results^16^. To get a better comparison with 2D theoretical simulations (Fig. 1), taking into account the scaling factor necessary to convert between the frequencies of the 2D structure and the 3D slab one (~1.325^16^), we obtain that the experimental spectral limit among the two ranges (1250 nm) is in normalized frequency values *a*/*λ* = 0.229, in almost a perfect agreement with the numerical simulations (*a*/*λ*~0.221). Residual deviations between theoretical and experimental spectral measurements must be attributed to a variation in the filling fraction introduced by the fabrication process and slight modifications in the slab thickness.

Having reliably distinguished between delocalized and localized modes, we now focus on the latter. As in other disordered systems^24^, two distinct types of localized states can occur in hyperuniform disordered networks^10,12,16^: (i) genuine Anderson-localized modes, which arise from strong disorder and multiple scattering throughout the structure and are not strictly confined to band-edge regions. These resonances reflect a disorder-driven transition from extended to localized states and are associated with Wigner–Dyson and Poisson statistics for delocalized and localized modes, respectively, as indicated by the light blue and orange regions in the left panel of Fig. 1b. (ii) defect-induced, tightly localized modes, which are confined to only a few cells^10,12,16^ and are spectrally located in the immediate vicinity of the band edges. These modes are promoted into the band gap of the corresponding periodic structure by the presence of hyperuniform disorder.

**Fig. 3:**
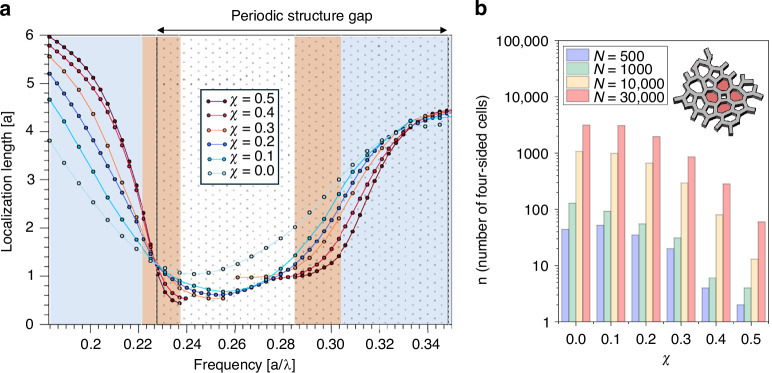
Disorder-dependent localization crossover and emergence of Lifshitz-like defect states in HuD photonic networks. **a** Localization length *ξ* of the HuD structure for different values of the stealthiness parameter *χ*, as a function of frequency. The white region in the center denotes the true photonic band gap of the hyperuniform disordered pattern at χ = 0.5, the value considered in this work, analogous to the right panel of Fig. 1b. This gap defines the spectral region associated with the orange and blue areas, corresponding to Poisson and Wigner–Dyson statistics. Upon decreasing χ, the band gap progressively shrinks and closes at χ = 0.2. The overlaid dotted pattern marks the gap of the corresponding periodic structure of the same filling fraction. **b** Histogram of four-sided topological defects (sketched in the inset) in different realizations of HuD networks varying *N* (500, 1000, 10,000, and 30,000) and for increasing values of *χ*, corresponding to decreasing degree of disorder

The two types of localized modes can be distinguished by analyzing their localization length at different degrees of disorder. In the HuD system, smaller values of the stealthiness parameter *χ* correspond to a higher amount of disorder^10^. By analyzing the localization length of HuD modes as a function of *χ*, we can discriminate between genuine Anderson-localized modes, whose localization length decreases with increasing disorder, and defect-induced modes, with localization length increasing with the degree of disorder present in the sample^10,24^. The results of this calculation, employed here as an operational and platform-specific method, are shown in Fig. 3a, where the *ξ* of the HuD structure is reported as a function of frequency for values of *χ* varying from 0 to 0.5 (more simulation results can be found in Supplementary Information, Fig. S2.1, showing *ξ* vs frequency for three sample sizes). The colors in the spectral windows are overlapped accordingly with the ranges investigated in Fig. 1. We observe that all *ξ* curves on the left side of Fig. 3a cross within a narrow spectral region centered around *a*/*λ* = 0.227, precisely defined by the lower PBG edge of the honeycomb periodic structure of the same filling fraction (highlighted as a dotted pattern in the figure). This coincidence is not arbitrary: in this frequency window, disorder no longer simply perturbs extended dielectric-band modes into Anderson-localized modes but starts promoting tightly localized resonances into the band gap. This is a first clue suggesting a transition from type (i) to type (ii). In the $a/{\lambda }\in$ (0.221–0.227) spectral range (highlighted in light orange), the modes can be labeled Anderson-localized (type (i)) as their *ξ* decreases a *χ* decreases (details on fluctuations and extra spectral range can be found in Supplementary Information, Fig. S2.2). We note that the nature of the Anderson modes above the upper band-edge of the photonic band gap of the HuD structure is rather distinct from that of the photonic modes below the lower band-edge. This difference is a quintessential of the HuD architecture employed here. Modes below the photonic band gap, the so-called “dielectric band”, have the electric field mostly concentrated in the dielectric fraction of the structure. Since the dielectric is structured as a connected network with walls of constant thickness and uniform trivalent connectivity, for the “dielectric-band” modes, the disorder manifests itself only as fluctuations in the coupling between electromagnetic resonances set up in the spatially uniform distribution of dielectric material. We note that the effect of the disorder is hence limited, and it results in a relatively small shift of the lower band gap edge compared to the periodic counterpart structure (a perfect honeycomb network of the same filling fraction). In sharp contrast, the photonic modes above the band gap, the so-called “air-band”, have most of the electric field concentrated in the air fraction of the HuD structure, i.e., inside the cells of the HuD network. While the dielectric fraction suffers only from positioning disorder, the air fraction of the network is subject to two concomitant hyperuniform disorder effects. On one hand, similarly to the dielectric fraction, the distance between the “air” cells varies across the sample (positional disorder), and, on the other hand, the cells have now different sizes and shapes (“form” disorder). This combined position, shape, and size disorder has a major influence on the upper band-edge modes, and as shown on the right panel of Fig. 1b, a much larger number of them are promoted inside the band gap of the corresponding periodic honeycomb structure.

For frequency above *a*/*λ* = 0.227, defect modes (type (ii)) of a special kind are being formed, with *ξ* increasing for smaller values of *χ*. These defects in HuD platforms come with a peculiar cavity-like feature due to their association with topological defect modes hosted by four-sided cells in the honeycomb-like hyperuniform network^10,12,16^ (see inset in Fig. 3b). With reference to the photonic band structure (left panel of Fig. 1b), the miniband inside the PBG consists of 12 defect modes matching precisely the number of four-sided-cell defects (highlighted in red in Fig. 1a) present in the structure. Four-sided cells modes are indeed invariably promoted inside the PBG of the correspondingly periodic honeycomb structure (corresponding to a frequency range $a/{\lambda }\in$ (0.227–0.349) and highlighted by the dotted area in Fig. 1b, right panel), displaying the tightest spatial localization of all the modes (Fig. 3a)^12^. Beyond the trend of ξ, the spectral and spatial peculiarities of four-sided-cells defects demonstrate that the introduction of a controlled amount of hyperuniform disorder (by changing χ) leads to a smooth crossing, occurring at the lower edge of the corresponding periodic honeycomb structure’s PBG, between modes of different nature. An additional analysis based on the band structure and mode profiles at the crossover in the localization behavior is reported in Supplementary Information, Fig. S3.1.

We argue that the modes associated with four-sided cells, which form a miniband within the photonic band gap, can be interpreted as the photonic analog of Lifshitz states known from electronic systems. In a hyperuniform disordered architecture, their peculiar topological origin implies a degree of predictability in their spatial location. To demonstrate this, we evaluate the occurrence of these states in the HuD sample of different sizes (*N* = 500, 1000, 10,000, and 30,000 points) for increasing values of *χ* (i.e., decreasing degree of disorder). The results, reported in Fig. 3b, show with extreme clarity that the number of four-sided-cell defects not only increases with the sample size (*N*), but also with disorder. In the strict statistical-mechanical sense, Lifshitz states are defined as exponentially rare, disorder-induced eigenmodes whose occurrence governs the asymptotic behavior of the density of states near a band-edge, giving rise to Lifshitz tails in the thermodynamic limit^29–32^. Demonstrating such states rigorously requires large-scale statistical simulations of the density of states over many disorder realizations and system sizes, as recently discussed for hyperuniform systems in ref. ^32^ While such an asymptotic analysis is beyond the computational scope of the present work, the localized band-edge modes observed here display the key phenomenological hallmarks commonly associated with Lifshitz physics: strongest spatial confinement, spectral proximity to the band-edge, and a disorder-induced origin (for a more detailed analysis of how the concentration of these states varies with sample size, see Fig. S3.2 of the Supplementary Information). Here and in the following, these states are referred to as “Lifshitz-like” states.

**Fig. 4:**
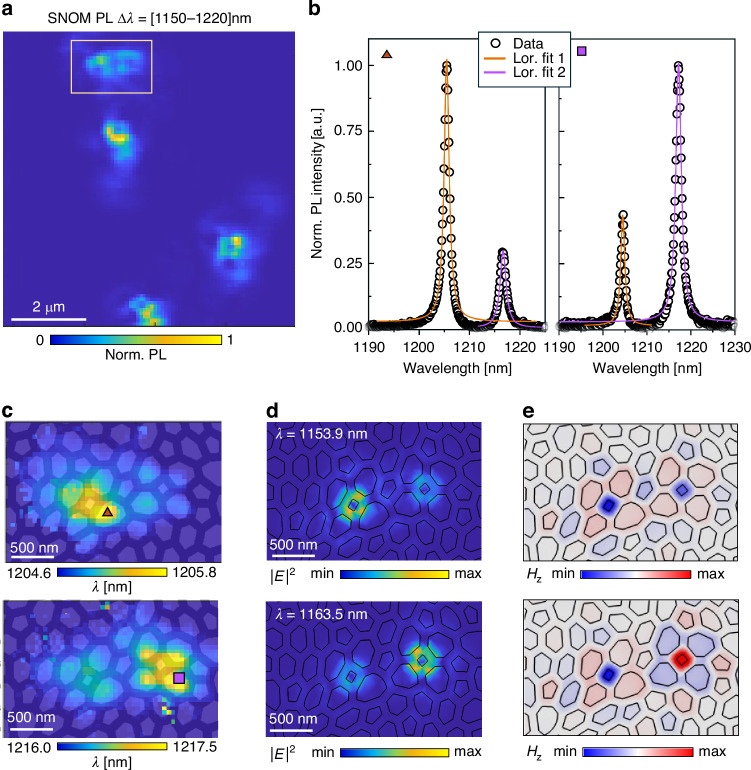
Hybridization of Lifshitz-like states into a photonic molecule. **a** SNOM PL map filtered around the PBG region where five Lifshitz-like states can be visualized. **b** SNOM PL spectra, each acquired on top of a single four-sided cell defect, and relative Lorentzian fits. The points of acquisition are highlighted by the orange triangle and purple square in the corresponding spectral shift maps, shown in (**c**). **d** FEM maps of the electric field intensity of the coupled Lifshitz-like defects. **e** Corresponding FEM maps of the out-of-plane magnetic field (*H*_*z*_), revealing bonding and antibonding behavior with locally symmetric and antisymmetric field distributions, respectively

In this scenario, one consequence of increasing the degree of disorder is that, due to their increased concentration, Lifshitz-like states localization is degraded, and weak spatial overlap can enable hopping-like transport, analogous to Mott’s variable-range hopping in electronic systems^43^. We therefore focus on the five Lifshitz-like states present in the fabricated samples, mapping their near-field spatial distribution with the hyperspectral PL map (filtered around ∆*λ* = [1150–1220] nm) reported in Fig. 4a. A pair of two spatially near Lifshitz-like defects can be spotted among the five modes (yellow rectangle in Fig. 4a).

We measure the local electric field intensity by evaluating, through Lorentzian fits, the tip-induced spectral shift^12^ on the two corresponding modes. Figure 4b reports the fitted PL spectra acquired in two different positions of the hyperspectral map, each corresponding to one of the two individual defects; we observe that the two peaks representing the two modes can be found with different weights on both defects. The morphological information given by the SNOM topography (see Supplementary Information, Fig. S3.3), combined with the spectral shift maps reconstructed by fitting the spectra in each pixel, allows us to establish that the signal of the two modes (Fig. 4c) is delocalized on both Lifshitz-like states. The spatial delocalization, together with the spectral splitting of ∆ = 11.4 nm, indicates a coupling between the two modes. We validate this observation through Finite-Element Method (FEM) simulations, whose maps of the electric field intensity are reported in Fig. 4d: despite the rigid spectral shift between theory and experiment (to be attributed to discrepancy between nominal and fabricated structural parameters like slab thickness and filling fraction) the FEM maps are in excellent agreement with the SNOM maps and the theoretical spectral splitting of ∆ = 9.6 nm is comparable with the experimental one. Moreover, Fig. 4e illustrates, through Finite-Element Method maps of the transverse magnetic field component (*H*_*z*_), the bonding and antibonding behavior characteristic of photonic molecules. To our knowledge, there is no prior experimental observation in electronic systems of hybridized Lifshitz tail states forming resolvable bonding–antibonding pairs. While theory predicts hybridization/splitting of localized levels in Lifshitz-type models^44^ and classic impurity-band studies imply overlap among tail states as concentration increases^45^, a direct experimental demonstration has never been accomplished. In photonic systems, previous studies of uncorrelated random media have reported hybridization only through local tuning or perturbative modifications^8,36^. Our results instead indicate that hyperuniform structures, while disordered, support an architecture-encoded predictability that enables controlled hybridization without post-fabrication tuning. This finding is extremely important since, when several of such localized states align spatially and spectrally, they can hybridize to form optical necklace states^3,7,35,36^, which act as cooperative pathways for light propagation through the disordered structure. Thus, photonic Lifshitz-like states serve as the building blocks for more complex phenomena, from photonic dimers to full necklace states, mediating sub-gap transmission in disordered photonic media. Our results thus provide the first clear evidence of this phenomenon and, given the special architecture upon which they rise in HuD, the aspect of predictability is then brought into the picture of these rare statistical events, making our findings unique and impactful.

## Discussion

We have demonstrated three fundamental advances in the understanding of light localization in HuD photonic networks, establishing principles that are broadly applicable to disordered photonics and wave systems beyond optics. First, we reported a direct experimental observation of level repulsion between delocalized modes in a HuD photonic structure. To enable this, we have employed a sub-diffraction hyperspectral approach based on autocorrelation analysis of SNOM data, which allowed us to detect modal overlap and level repulsion without relying on traditional level-spacing statistics. Detecting this phenomenon establishes delocalized HuD modes within the same universality classes as chaotic quantum systems, offering a rigorous benchmark for identifying true delocalization. At the same time, it highlights the idea that delocalized HuD resonances don’t appear at arbitrary frequencies but form a statistically correlated, stable spectrum, unlike Anderson-localized ones, which can pile up unpredictably. While the coexistence of different localization regimes in correlated disordered photonic systems has been anticipated by previous theoretical and numerical works, our results provide direct experimental access to these regimes in hyperuniform disordered photonic slabs, phenomenologically differentiating them from Anderson-localized modes. Rooted in four-sided topological defects, Lifshitz-like states in the HuD photonic network are here revealed not as rare statistical anomalies, but as localized states with spatial and spectral location predictable at the design stage. Finally, we uncovered the hybridization of Lifshitz-like states into photonic molecules, which represent the first step toward the creation of chains of hybridized localized random modes, better known as necklace states^3,7,35,36^, with significant implications for random lasing and photon transport. Our findings establish a new regime of photonic disorder, where localization, spectral correlation, and mode coupling coexist in a single platform, which is in principle controllable at the design level as well as multifunctional (supporting different modal regimes in the same structure). The ability to design and reproducibly implement localized and interacting modes through architecture-encoded features suggests multiple application pathways. While Lifshitz-like modes in HuD could enforce on-chip light confinement, spectral level repulsion implies a stability which can provide a new degree of control over disorder-enabled transport, relevant for applications from random lasing^46^ to multiplexing and optical filtering^47^, where correlated spectra rather than isolated resonances are essential. Moreover, coupled localized states are relevant for neuromorphic or quantum photonic device^48^. The findings presented in this study provide essential and novel insights into light localization in complex photonic architecture structures and establish a general framework where structural disorder becomes a potential platform for wave-based technologies across optics, acoustics, and beyond.

## Materials and methods

### Theoretical design and simulations

HuD networks were generated within a square computational domain under periodic boundary conditions. A characteristic length scale $a=L/\sqrt{N}$ is introduced in analogy with the photonic crystals lattice constant, ensuring a uniform point density of $1/{a}^{2}$, where $N$ denotes the number of scattering centers within the supercell. The HuD point pattern was produced following the protocol reported in ref. ^10^, ensuring suppressed long-range density fluctuations and vanishing Fourier components within a defined wavenumber cutoff. The photonic band structure and density of states under transverse-electric (TE) polarization were calculated using the eigenmode expansion software MPB^49^ for a supercell of side $a\sqrt{N}$ using the associated high-symmetry points of the resulting Brillouin zone (of characteristic size $2{\pi }/(a\sqrt{N})$). Additional 3D finite-element method (FEM) calculations were performed to model the electric field distribution of coupled Lifshitz-like states and to validate experimental observations of hybridized “photonic molecule” modes. FEM simulations were performed by using the commercial software COMSOL^50^; the 3D membrane was modeled by extruding the two-dimensional theoretical design (Fig. 1a) along the vertical direction to form a slab of thickness *t* = 220 nm. Only one vertical half of the membrane was simulated by imposing a perfect magnetic conductor (PMC) boundary condition at the mid-plane of the slab, thereby selecting symmetric solutions. Perfectly matched layers (PMLs) with a thickness of 2*a* (with *a* = 380 nm defined as the characteristic length scale of the hyperuniform disordered network) were applied on all four in-plane boundaries, as well as above an air layer of thickness *7t*/2 = 770 nm on top of the membrane. In all simulations, a refractive index of 3.4 was used.

### Sample fabrication

The optically active photonic slabs were based on GaAs membranes incorporating a dense layer of self-assembled InAs quantum dots (QDs), with emission spanning 1100–1300 nm. The epitaxial stack consisted of a 220 nm GaAs layer grown atop a ~3 µm AlGaAs sacrificial layer to allow membrane release. Molecular beam epitaxy was employed to realize the heterostructure, while the HuD patterns were transferred onto the membrane using electron-beam lithography, followed by reactive-ion etching and a selective removal of the AlGaAs sacrificial layer^12,16,17,35^.

### Experimental characterization

Near-field optical measurements were performed using a commercial SNOM in illumination/collection geometry. A chemically etched dielectric fiber probe was raster-scanned tens of nanometers above the sample surface, simultaneously collecting topographic and optical signals. Local excitation was provided by a 785 nm diode laser, coupled through the fiber tip, while the emitted PL was dispersed by a spectrometer and detected by a cooled InGaAs array. The system achieved a lateral spatial resolution of ~250 nm and a spectral resolution of 0.11 nm, enabling reconstruction of hyperspectral maps of the local density of states^12,16,17,35^.

## Supplementary information

Supplementary information for spectral level repulsion and Lifshitz-like states in hyperuniform disordered photonic networks

## Data Availability

The data that supports the findings of this study are available from the corresponding author upon reasonable request.
