# Supplementary material for: Spectral level repulsion and Lifshitz-like states in hyperuniform disordered photonic networks
Source: Light Sci Appl. 2026 May 20;15:245. doi: 10.1038/s41377-026-02335-0 (PMC13190682; doi:10.1038/s41377-026-02335-0)
Supplement: Supplementary file 1 — Supplementary information for spectral level repulsion and Lifshitz-like states in hyperuniform disordered photonic networks [file 41377_2026_2335_MOESM1_ESM.docx]

**Supplementary Information for**

**Spectral Level Repulsion and Lifshitz-like States in
Hyperuniform Disordered Photonic Networks**

Nicoletta Granchi^1*^, Gabriele Calusi^1^, Kris Stokkereit^2^, Matteo Lodde^3^, Camilla Gonzini^1^, René P.J. van Veldhoven^3^, Andrea Fiore^3^, Marian Florescu^4^ and Francesca Intonti^1^

*^1^Department of Physics and Astronomy and LENS, University of Florence, Italy‎*

*^2^ Advanced Technology Institute and Department of Physics, University of Surrey, UK*

*^3^Department of Applied Physics and Science Education, Eindhoven University of Technology, The Netherlands*

*^4^ Optoelectronics Research Centre, University of Southampton, UK*

^*^corresponding author: nicoletta.granchi@unifi.it

**S1. The autocorrelation function technique**

In this Section we describe the procedure adopted in our work, (as well as in previous studies in Ref. [1,2]) to evaluate the autocorrelation of near-field maps. As shown in Fig.S1, we first defines the local near-field spectrum $I(\lambda)$ at each spatial position, and then evaluate the intensity autocorrelation of every spectra:

$$\int I\left( \lambda' \right)I\left( \lambda^{'}-\Delta\lambda\right)d\lambda'$$

As shown in Fig.S1.1, the autocorrelation of a single near-field spectrum presents some peculiar features: i) the narrow spike centered at 0, due to the self-correlation of the individual modes, ii) small values of the autocorrelation function around the self-correlation peak, and iii) different correlation resonances at larger $\Delta\lambda$*.* By averaging this quantity over all the pixels in the SNOM map, we get: $R\left( \Delta\lambda\right)=\left\langle\int I\left( \lambda' \right)I\left( \lambda^{'}-\Delta\lambda\right)d\lambda' \right\rangle$.

In parallel, we evaluate the autocorrelation of the average spectrum $\left\langle I\left( \lambda\right) \right\rangle$, $R_{0}=\int\left\langle I\left( \lambda' \right) \right\rangle\left\langle I\left( \lambda^{'}-\Delta\lambda\right) \right\rangle d\lambda'$, which accounts for the uncorrelated contributions. The latter are subtracted from $R\left( \Delta\lambda\right)$ to obtain the autocorrelation function:

$$Rc(\Delta\lambda) = R(\Delta\lambda) - R_{0}(\Delta\lambda)$$

i.e. the quantity reported in Fig. 2c. The $R_{c}(\Delta E)$ quantity encodes information about microscopic spectral correlations, *including level repulsion*. In the language of Random-Matrix Theory, Wigner–Dyson statistics implies that the two-level correlation function vanishes at zero separation, reflecting level repulsion between eigenvalues. In an *idealized* system of infinitely sharp lines, this would translate into a suppression of correlations at$\Delta\lambda\approx0$in $R_{c}(\Delta\lambda)$. However, as shown in Ref. [1] , each discrete level has a finite Lorentzian width, and the self-correlation of these Lorentzians produces a strong peak in both $R(\Delta\lambda)$and $R_{0}(\Delta\lambda)$at $\Delta\lambda=0$. When one forms $R_{c}(\Delta\lambda)$, the ideal dip from level repulsion is therefore not observed as a zero at $\Delta\lambda=0$, but instead emerges as a characteristic shoulder or minimum at a finite $\Delta\lambda$, whose position is set by the interplay between the mean level spacing and the Lorentzian linewidth. In contrast, for a spectrum with Poisson statistics (no level repulsion), $R_{c}\left( \Delta\lambda\right)$does not develop such a short-range suppression; it is dominated by the central peak only. Thus, in this established autocorrelation approach, it is precisely the presence or absence of a shoulder in $R_{c}\left( \Delta\lambda\right)$at small but finite $\Delta\lambda$ that is used as the experimental signature of level repulsion.

**References**

1. Intonti, F., Emiliani, V., Lienau, C., Elsaesser, T., Savona, V., Runge, E., Zimmermann, R., Nötzel, R. & Ploog, K. H. Quantum Mechanical Repulsion of Exciton Levels in a Disordered Quantum Well. *Phys. Rev. Lett*. 87, 076801 (2001).
2. von Freymann, G., Kurtz, E., Klingshirn, C. & Wegener, M. Statistical analysis of near-field photoluminescence spectra of single ultrathin layers of CdSe/ZnSe. *Appl. Phys. Lett* 77, 3 (2000).

**
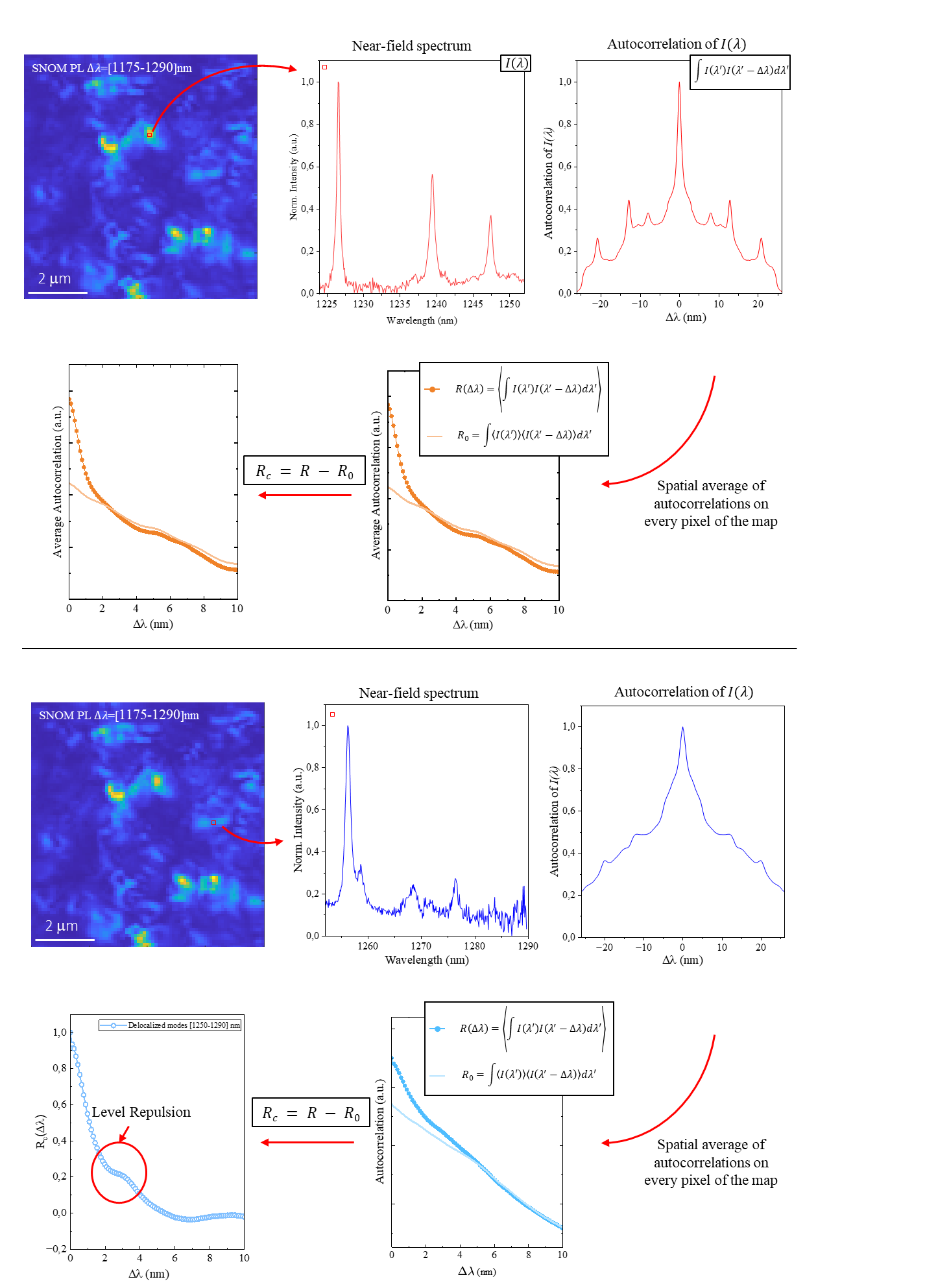
**

**Figure S1.1:** Schematics of the autocorrelation technique applied to a SNOM measurement focusing on localized modes (upper panel) and delocalized modes (lower panel).

**
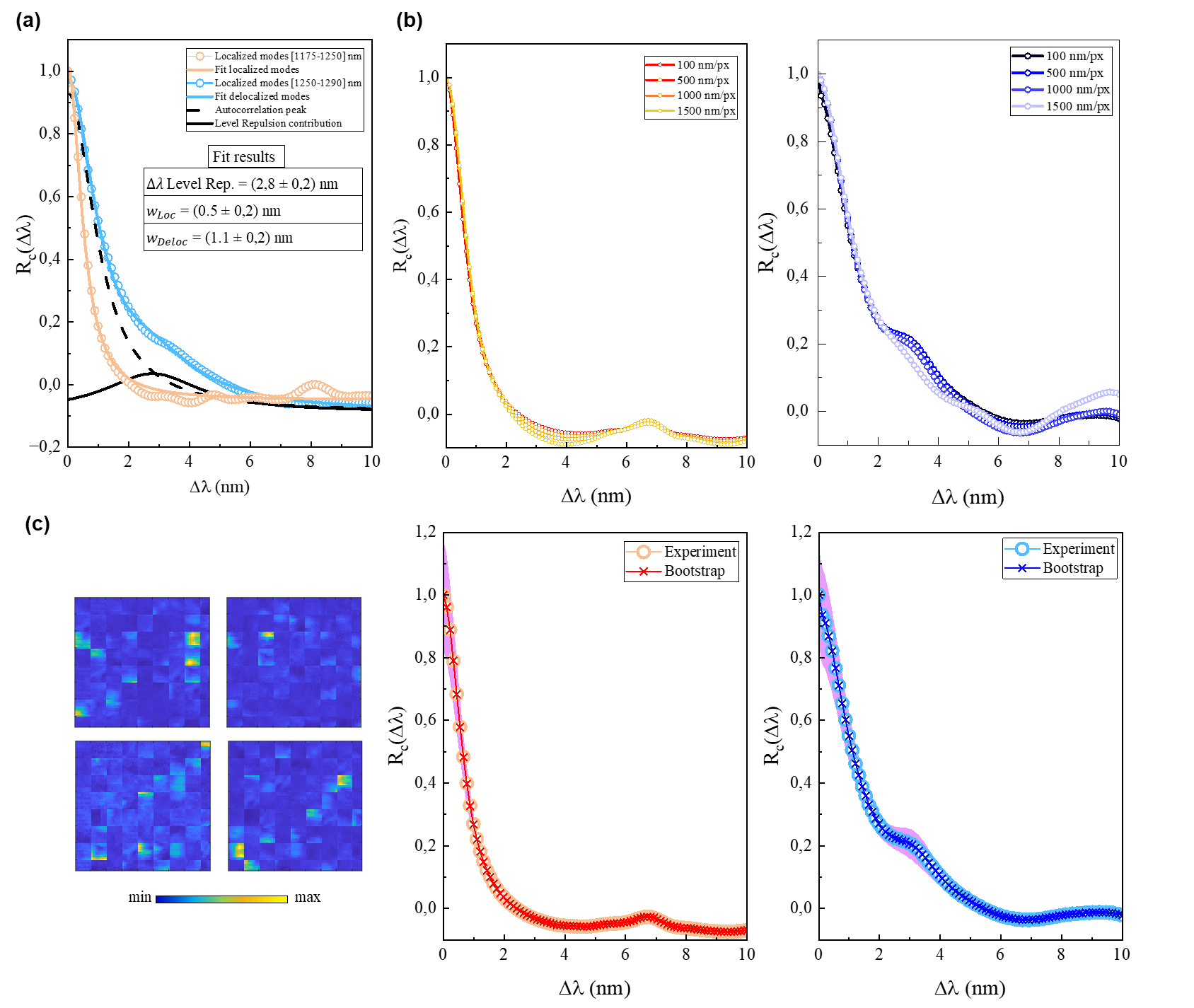
Validation of the Autocorrelation Analysis: Reproducibility, Statistics, and Resolution Effects**

**Figure S1.2:** (a) Autocorrelation function evaluated from a SNOM measurement performed on a second realization of HuD sample obtained from a different crop of the original design, i.e. with the same structural parameters (length scale $a$ = 380nm, wall thickness $w=0.34a$ and stealthiness $\chi=0.5$). The fitted contribution of the level repulsion is found at $\Delta\lambda$= (2.8$\pm$0.2) nm, consistent with value found for the reference sample ($\Delta\lambda$= (3.1$\pm$0.2) nm). (b) $R_{c}\left( \Delta\lambda\right)$recalculated on the design considered in main text after progressively increasing the effective spatial sampling step of the original near-field map from 100 nm/px to 1500 nm/px, emulating controlled spatial coarse-graining. The autocorrelation curves remain qualitatively unchanged as long as the sampling length is smaller than or comparable to the characteristic spatial extent of the optical modes. Only when the spatial step approaches or exceeds the modal size ($\sim$1000 nm) does the shoulder associated with level repulsion progressively weaken due to loss of spatial information. (c) Two-dimensional bootstrap analysis of the original SNOM map, performed by randomly resampling square blocks of size 10 px × 10 px (corresponding to 100 nm/px) with replacement and reconstructing randomized maps of equal size. For each of 1000 independent realizations (4 of which are reported as an example on the left of the figure), $R_{c}\left( \Delta\lambda\right)$is computed in the localized and delocalized spectral windows. The curves are ensemble-averaged, and the standard deviation at each $\Delta\lambda$is shown as a shaded region, providing confidence intervals.

**
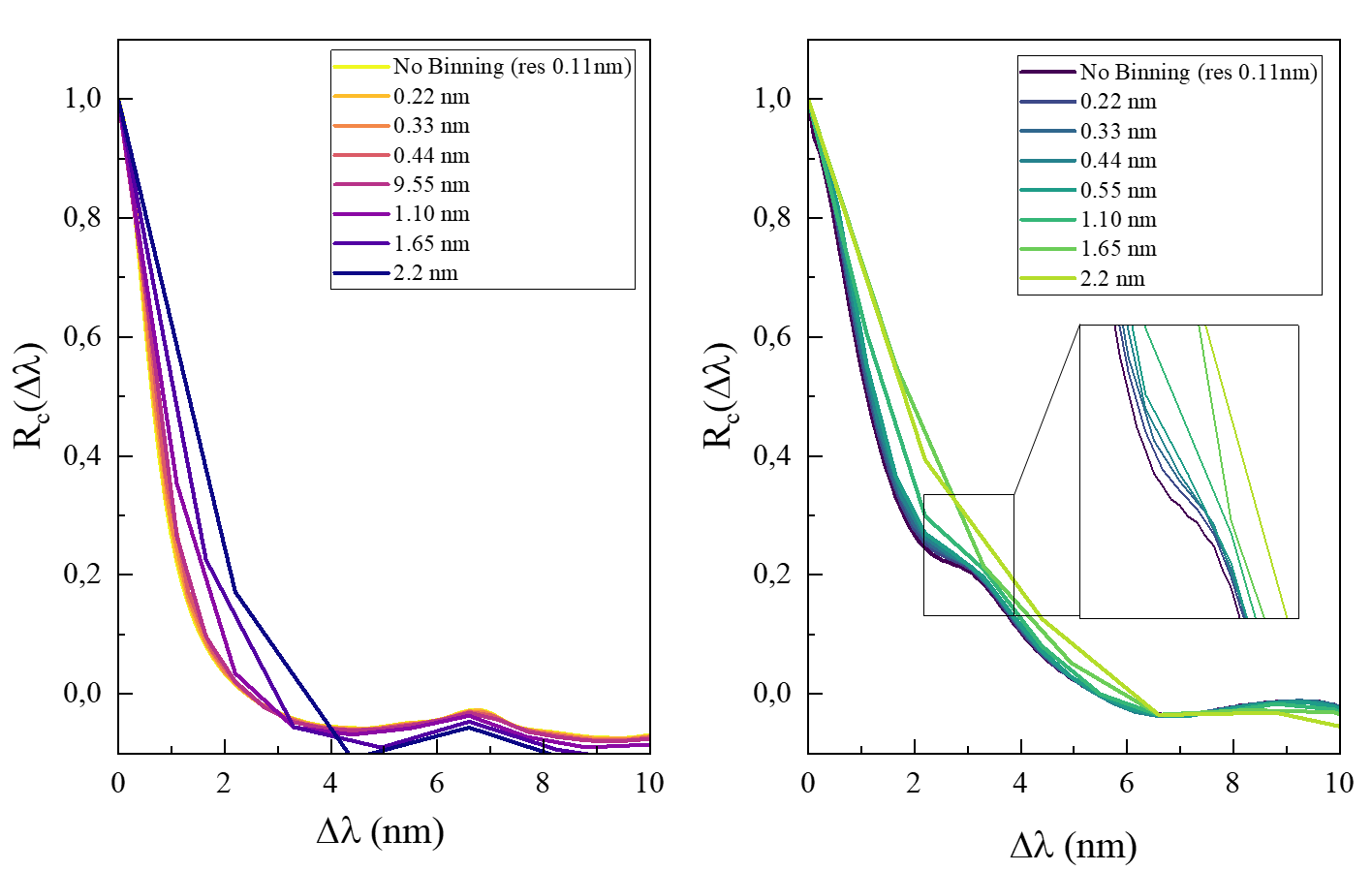
Figure S1.3:** Effect of spectral resolution degradation on the autocorrelation function. $R_{c}\left( \Delta\lambda\right)$is calculated within the two spectral windows (localized and delocalized modes) after progressively increasing the spectral binning applied to the measured spectra. The un-binned spectral resolution is 0.11 nm px^-1^; binning over $N$ pixels corresponds to an effective spectral resolution of $N\times0.11$nm (see legend). As the spectral resolution is degraded, the shoulder associated with level repulsion is progressively smoothed and eventually suppressed, while its position remains unchanged at moderate binning.


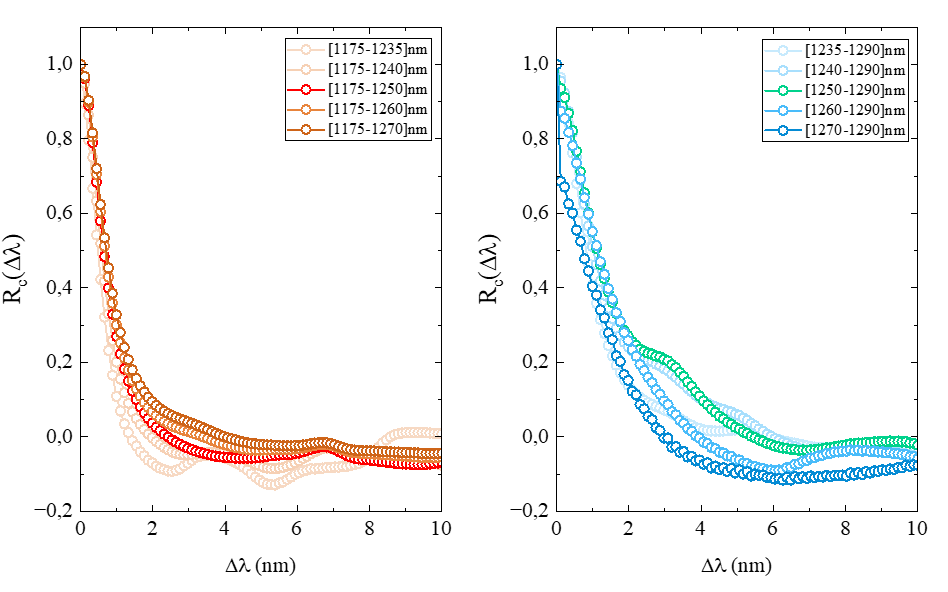


**Figure S1.4:** $R_{c}\left( \Delta\lambda\right)$ calculated in two spectral windows by systematically changing the upper (for localized modes) and lower bound (for delocalized modes). The shoulder in $R_{c}$ of delocalized modes, which is associated with level repulsion, progressively weakens and eventually disappears when the lower edge of the delocalized spectral window is shifted toward shorter wavelengths (down to 1235 nm). Analogously, with a shift above 1250 nm in the upper bound of the calculation window for localized states, the shoulder of level repulsion appears. The curves corresponding to the correct spectral windows (the same of Fig. 3c in the main text), are highlighted in red and green.

**S2. Localization length**

**
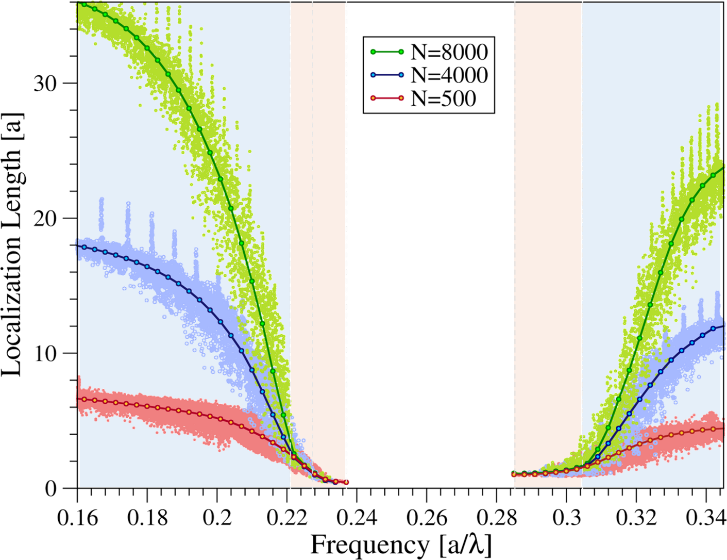
**

**Figure S2.1:** Localization length $\xi$ of the HuD structure with stealthiness parameter $\chi$=0.5, evaluated as a function of frequency for three sample sizes, i.e. different number of points in the original stealthy hyperuniform pattern, N=500, 4000, and 8000. The color code for the various spectral regions is the same as in the main text with light blue denoting the spectral region with mode statistics best fitted to a Wigner-Dyson distribution, the light brown region is associated with localized modes best fitted to a Poisson distribution. For localized modes with Poisson level spacing statistics, the localization lengths $\xi$ is insensitive to the system size, hence confirming their localized nature.


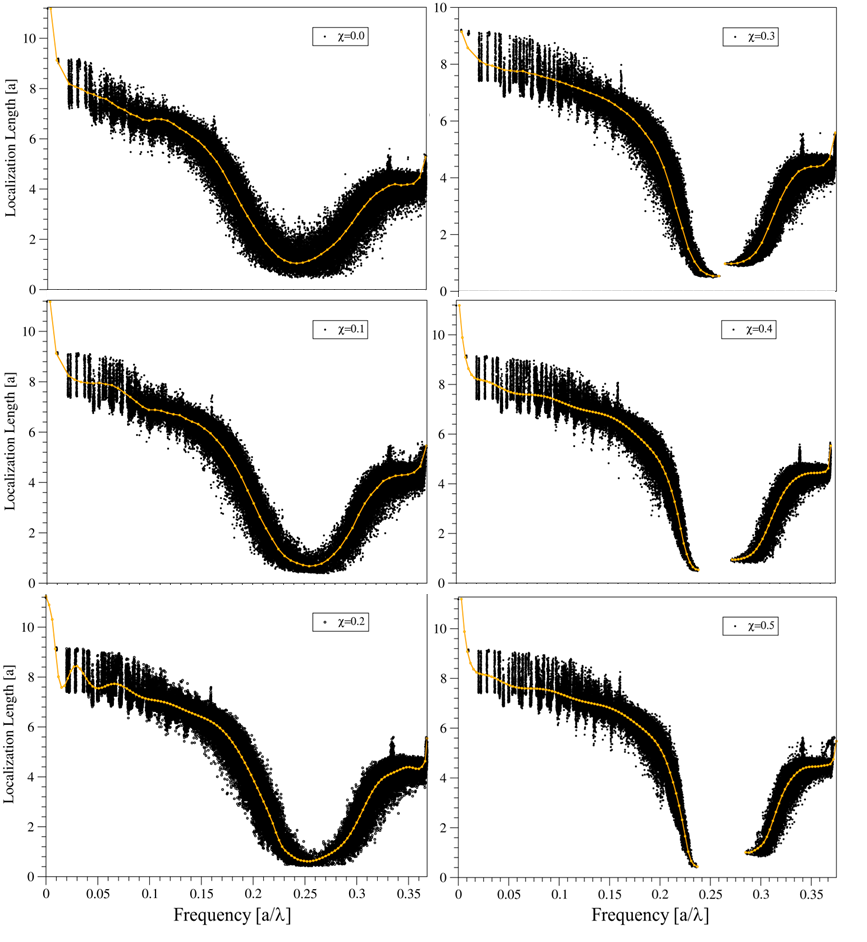


**Figure S2.2:** Localization length $\xi$ of HuD structures with stealthiness parameter varying from $\chi$=0 to $\chi$=0.5. The calculations are done for an ensemble of 50 *N*=500-point samples and 17 *k*-points per sample. The black dots represent the raw data, and the yellow curve is a spline fit of the average value 〈 $\xi$*/a* 〉 in a narrow frequency range ($[a/]$ = 5 × 10^−3^) around the frequency $a/$ considered. Despite the completely different morphologies of the samples corresponding to different χ values, all HuD structures studied exhibit a remarkably consistent spectral behavior of the localization length. In particular, the frequency range over which the localization length is reduced remains notably similar, pointing to a robust underlying mechanism governing light localization in HuD photonic.

**
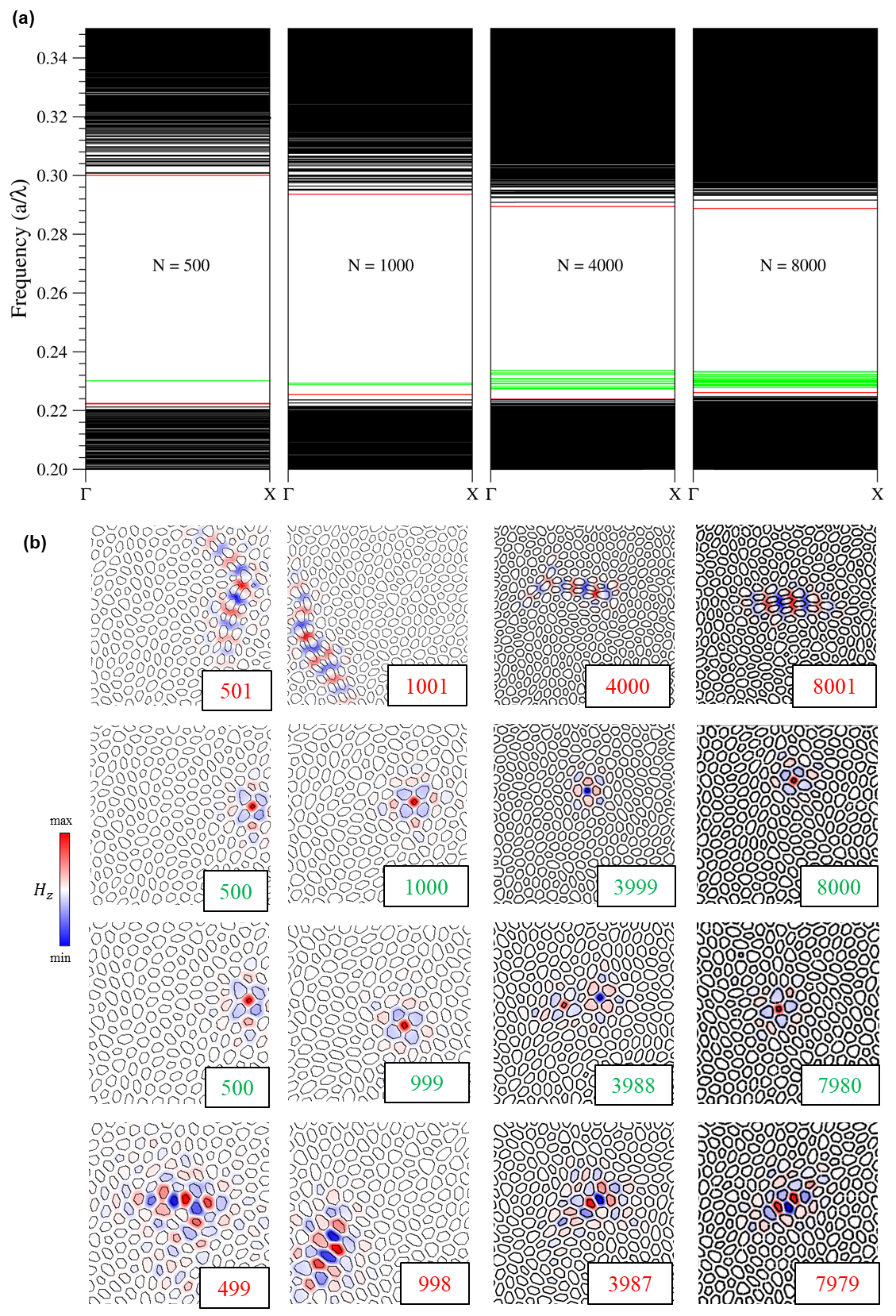
S3. Lifshitz-like state**

**Figure S3.1:** **(a)** Band structure for HuD networks with χ=0.5 and varying number of cells, N=500, 1000, 4000, 80000. The green modes are associated with Lifshitz-like states pinned on 4-sided defect cells. Their number perfectly matches the number of 4-sided cells identified in the respective pattern and they correspond to modes [N-n+1, N-1], with N the total number of cells and n, the number of 4-sided cells in the hyperuniform network, respectively. In red we show the Anderson localised air- and dielectric- modes in the immediate neighbourhood of the Lifshits-like states. (b) Magnetic field, $Hz$ profile, for the first Anderson localized modes above (air modes) and below (dielectric modes) the bandgap (red labels) and Lifshitz-like state (green labels) for a hyperuniform network with $\chi$=0.5 of increasing size: N=500, 1000, 4000 and 8000. This comparison shows that the narrow miniband above the crossover frequency persists across different system sizes and is systematically defined by the boundary between Anderson localized modes spanning a few cells and consistent with interference-driven Anderson localization and Lifshitz-like modes localized on four-sided cells predicted by the hyperuniform geometry. The corresponding mode profiles clearly demonstrate the very different nature of the localization properties confirming the presence of a well-defined crossover in localization behavior across a narrow spectral range.

**
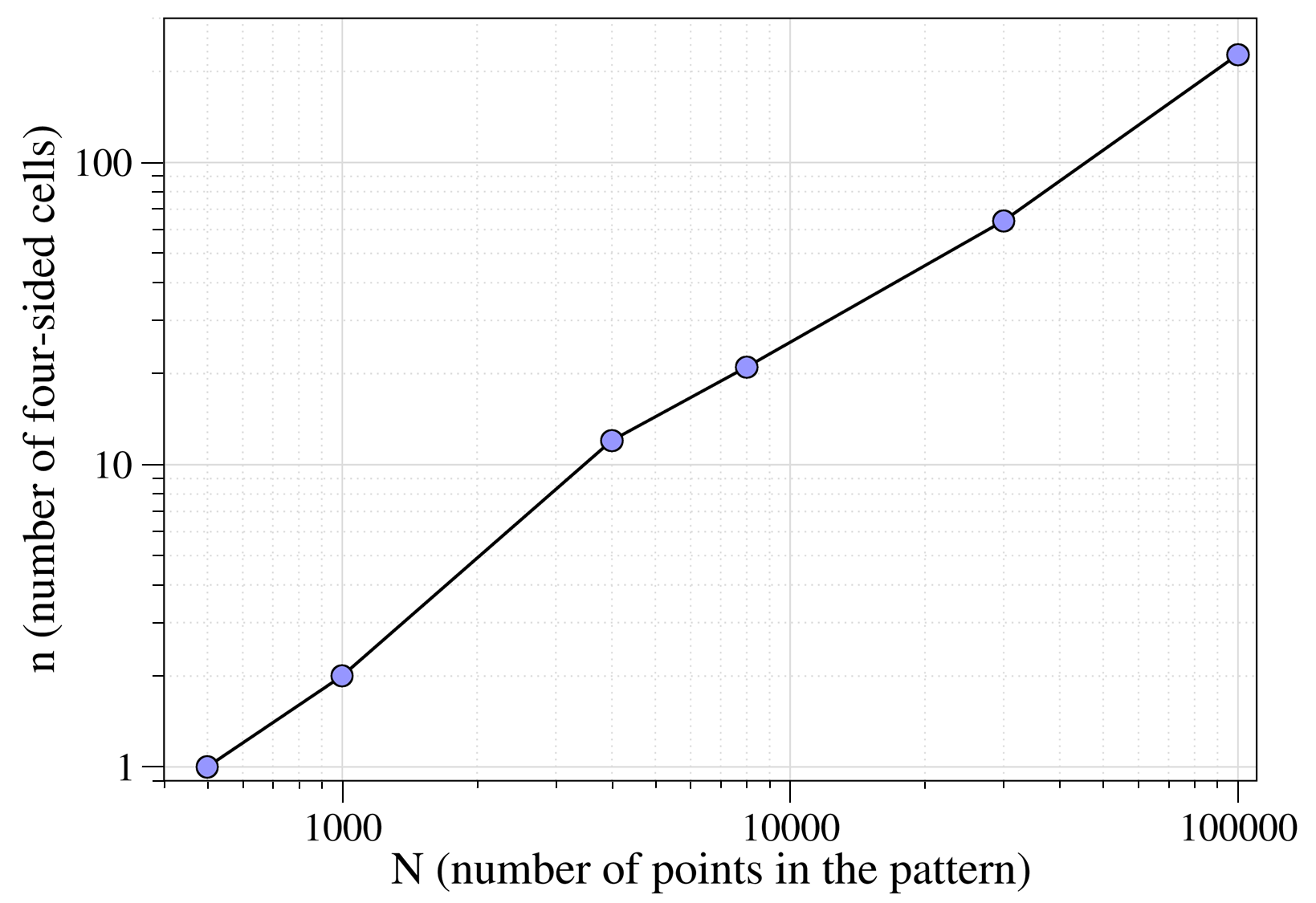
**

**Figure S3.2:** Number of four-sided cells in HuD structures with stealthiness parameter $\chi$=0.5, as a function of the number of points in the original (not cropped) stealthy hyperuniform pattern. For $\chi$=0.5, four is the smallest number of sides per cell in the HuD network, and all these four-sided cells act as defects that trap modes promoted within the band gap of the corresponding honeycomb periodic structure**.**


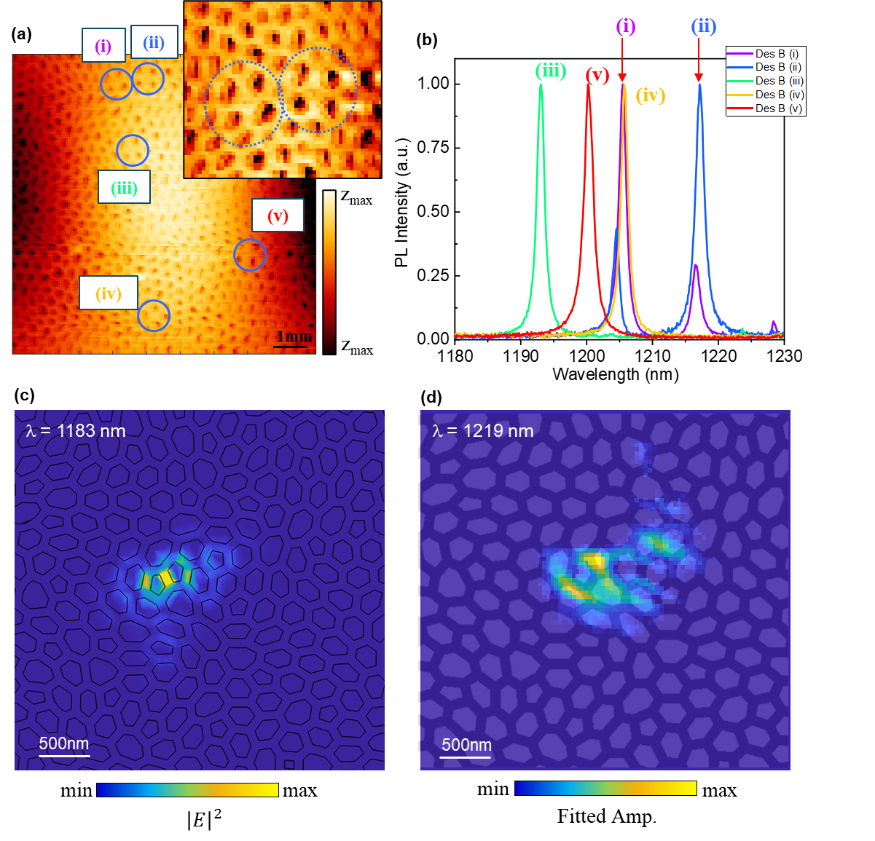


**Figure S3.3: (a)** Scanning Near-field Optical Microscopy (SNOM) topography of the sample, highlighting with circles the five Lifshitz states. The inset is a zoom of the topography displaying the couple (i)-(ii) investigated in Fig. 3 of the main text. **(b)** PL spectra acquired in correspondence of the points of maximum PL intensity of each Lifshitz mode. **(c)** Finite Element Method (FEM) map of the electric field intensity of the first Anderson localized mode at the photonic bandgap lower edge. **(d)** SNOM PL fitted amplitude map of the first Anderson localized mode overlapped with the HuD design. The fit is performed with a Lorentzian lineshape for every spectrum of the hyperspectral map as described in the text.
